# Supplementary material for: Exploring the non-linear association of daily cigarette consumption behavior and food security- An application of CMP GAM regression
Source: PLoS One. 2025 Jul 17;20(7):e0328109. doi: 10.1371/journal.pone.0328109 (PMC12270119; doi:10.1371/journal.pone.0328109)
Supplement: Attachment — Submitted filename: Response to Reviewers.docx [file pone.0328109.s001.docx]

Dear Editor-in-Chief,

We, the authors of PONE-D-24-42111 titled “Exploring the non-linear association of daily cigarette consumption behavior and food security- An application of CMP GAM Regression,” appreciate the reviewer’s comments, who managed the time to examine this manuscript thoroughly and provided us with valuable insights. These comments helped us immensely in revamping the manuscript, and the manuscript has been revised accordingly. We believe the contents and clarity of this manuscript are much improved in the revised version.

Below are the responses to the reviewers’ comments. The source file (.docx) of the revised manuscript was attached in two forms: ‘with changes marked’ and our ‘revised manuscript.’

**Additional Editor**

The responses to specific comments are as below

1. Although this study used the data from NHIS, please also clarify the sampling method and the inclusion and exclusion criteria for the analyses

**Response**

Thank you for your insightful comment. We have incorporated details on both the sampling method and the inclusion and exclusion criteria for the survey (in lines 140-158), as well as the inclusion and exclusion criteria for our analysis (in lines 166-171). These updates have been added to the Methods section for clarity. We appreciate your feedback in strengthening the transparency of our study.

**Sampling method and the inclusion and exclusion criteria for the survey**

The NHIS is an annual household survey representing the civilian non-institutionalized US population across the 50 states and the District of Columbia. It collects data from individuals residing in households and noninstitutional group quarters, including homeless shelters, rooming houses, and group homes. Individuals temporarily living in student dormitories or temporary housing are sampled within their permanent households.(1) The survey excludes those without a fixed household address, active-duty military personnel and civilians on military bases, residents of long-term care institutions (such as nursing homes and chronic care hospitals), individuals in correctional facilities, and U.S. nationals living abroad.(1) However, civilians residing with military personnel in non-military housing remain eligible for inclusion. The NHIS employs a geographically clustered sampling design to select dwelling units, ensuring national representativeness.(1) Based on the 2010 decennial census, the sampling process partitions the United States into 1,689 geographic areas, comprising counties, county equivalents, or contiguous county groups, ensuring they remain within state boundaries.(1) The sample is structured in a way that monthly data is nationally representative.(1) Usually, interviews for the NHIS are conducted in-person modules and followed up via telephone. For over 50 years, the U.S. Census Bureau has sent interviewers to American homes to inquire about various health topics. Data collection is continuous, spanning from January to December each year, allowing for timely and comprehensive health surveillance.(1)

**Inclusion and exclusion criteria for the analysis**

The inclusion and exclusion criteria for this study were determined based on the variables of interest and their alignment with the research objectives. From the 27,651 responses, (n=1,267) data were excluded as missing food security information. Additionally, non-smokers were excluded from the study (n=25,565). Out of 27,651 respondents, only 819 met the inclusion criteria based on the specified variables. The final selection was guided by the study's research objectives to ensure relevance and accuracy in the analysis.

2. please report the ethic statement for the study which the data collected from.

   **Response**

   Thank you for your valuable suggestion. We have now included the ethics statement for the study, specifying the ethical considerations related to data collection. This update has been added to the Methods section in lines 369-376 for clarity. We appreciate your feedback in enhancing the completeness of our manuscript.

**Ethics statement**
The Research Ethics Review Board (ERB) of the National Center for Health Statistics (NCHS) approved the content and methods of the National Health Interview Survey (NHIS) to protect the study participants.(2) Prior to taking part in the NHIS study, all participants provided their verbal consent. Potential NHIS respondents were informed about their rights regarding survey participation and were assured of the confidentiality of their responses, as well as the protection of their data.(3) All data in the publicly available dataset are fully anonymized prior to release. All authors declare they have no competing interests.

**Reviewer- 2**

3. The inclusion of too much nonlinearity will reduce the interpretability of the model. Does the author have any countermeasures for this?

   **Response**
   We appreciate the reviewer’s concern regarding model interpretability in the presence of excessive nonlinearity. To address this, we have carefully balanced linear and nonlinear components in our CMP GAM model. Specifically, we included a linear term in the model to account for the overall trend while allowing nonlinearity to capture subtle deviations where appropriate.
4. This is a cross-sectional data. In addition to subgroup analysis, are other bias control measures taken?

   **Response**
   We highly appreciate the reviewer’s thoughtful feedback regarding bias management in a cross-sectional study. While our current analysis did not explicitly account for confounders or mediation effects, we acknowledge their potential influence on our findings. In this study, we primarily focused on subgroup analysis to explore variations in associations. However, we recognize the importance of addressing confounding and mediation to better understand the pathways through which the exposure influences the outcome, and we mentioned this accordingly in the limitations section of the paper in lines (545 – 548). As part of our future research, we will incorporate causal mediation analysis and confounder adjustment techniques, such as stratification and regression-based controls, to assess these relationships comprehensively. We sincerely appreciate this valuable feedback and will carefully consider these aspects in subsequent work.

   **Added details to the limitations section**

“Additionally, this study did not conduct confounding or mediation analyses, which could have provided a deeper understanding of the pathways through which the exposure influences the outcome. Future research should consider incorporating these analyses to assess these relationships more comprehensively.”

**Reviewer- 3**

5. Line 49-71, the background of reason of food insecurity to increase or decrease were not enough to support your hypothesis.

   **Response**
   Thank you for your valuable suggestions. We have incorporated the supporting literature reviews and evidence into the specified section. The relevant updates can be found between line numbers 49 and 76 and mentioned below.

“Previous research has shown that socioeconomic factors, including food security, are closely linked to smoking behavior. An earlier study among young adults observed that smoking prevalence is significantly higher for young adults who reported being food insecure.(4) The effect of food insecurity on smoking behavior is particularly noticeable among low-income and marginalized populations. Low-income adults experiencing food insecurity have been found to have a disproportionately high prevalence of cigarette smoking.(5) The impact of food insecurity on smoking behavior is multifaceted. Studies have shown that food insecurity is a barrier to smoking cessation.(6) Previous studies have discovered that facing food insecurity is associated with a higher likelihood of starting smoking for those who do not smoke.(7) Furthermore, Baek et al. noted that shifts from food security to insecurity were associated with almost a twofold rise in the likelihood of smoking behavior.(8) Individuals experiencing food insecurity may use smoking as a coping mechanism to manage stress and anxiety associated with their precarious food situation.(9) Canales et al. (2015) delineated that worrying about running out of food is an important indicator of food security and has direct causal implications on one’s perceptions of stress or anxiety. Whereas cigarette smoking is a common way to relieve tension and stress,(10) suggesting an association between the intensity of smoking and food insecurity. Individuals experiencing food insecurity may turn to smoking as a way to manage stress or suppress appetite, which can further complicate efforts to quit smoking.(11) Additionally, financial constraints can lead to prioritizing immediate relief through smoking over long-term health investments.(12) Psychological distress exacerbated by food insecurity may promote smoking for stress relief, creating a cycle that hinders cessation.(4) Moreover, a dose-response relationship has been observed between tobacco product use and levels of food insecurity, indicating that higher levels of smoking-induced financial deprivation can worsen food insecurity.(13) Nicotine intake reduces appetite and suppresses hunger,(14) which increases the likelihood of smokers using smoking as a coping mechanism to reduce hunger related to food scarcity. This dynamic exacerbates health disparities and significantly burdens public health systems. Taking these into account, there is a growing recognition of the need for more comprehensive assessments to understand the dynamics between tobacco use and food insecurity.”

6. line 72-85, some sentences need to be segment, they are quite difficult to understand. The writing structure seems like evidences stacks, however, the reason of non-linear were not introduced were not present well.

   **Response**
   We appreciate your insightful feedback. We have revised the sentences in lines 77-89 to improve clarity and readability. The writing structure has been adjusted, and a new section has been added to better introduce and present the reasons for the non-linear approach in lines 90 and 104. We hope these changes address your concerns and enhance the overall coherence of the section.

   **Revised paragraph**

   Numerous statistical models have been utilized in research studies to explore the association between food insecurity and smoking behaviors. In most of the research studies, logistic regression analysis was used to examine how food insecurity is associated with smoking status. (6,10,15) Longitudinal logistic and Tobit regressions analyzed the link between food security and smoking behaviors in low-income women, focusing on the longitudinal impact of food insecurity on smoking habits.(16) A prior study used multinomial logistic regression to assess and characterize the relationship between food security and tobacco product use.(17) Another study utilized multinomial logistic regression to examine the association between transitions of food insecurity with smoking cessation and shifts in the consumption pattern over two years among adults.(18) However, most studies only considered a linear association between food security and cigarette smoking behavior. Nevertheless, the non-linear nature of the association requires further investigation. To our knowledge, there is no study that has been conducted to capture the non-linear association between those variables using an appropriate count data model.

**Reasons for incorporating non-linearity**

Considering a non-linear association between food security and cigarette smoking behavior is important for several reasons:

**Complex Relationships**: The relationship between food security and smoking behavior may not be straightforward. Non-linear models can capture more complex interactions and thresholds that linear models might miss. For example, the impact of food insecurity on smoking behavior might be more pronounced at certain levels of food insecurity.(4)

**Bidirectional Influences**: Food insecurity and smoking behavior can influence each other in multiple ways. For instance, food insecurity might lead to increased stress and smoking as a coping mechanism, while smoking can exacerbate financial strain, worsening food insecurity.(10)

**Policy and Intervention Design**: Understanding non-linear relationships can help in designing more effective policies and interventions. For example, interventions might need to be targeted differently at various levels of food insecurity to be effective.

**Literature Contribution**: Incorporating non-linear associations enriches the literature by providing a more nuanced understanding of the dynamics between food security and smoking. It challenges the oversimplified view of linear relationships and encourages further research into the underlying mechanisms.

Overall, by considering non-linear associations, researchers can uncover more detailed insights and develop more targeted strategies to address the intertwined issues of food insecurity and smoking behavior.

7. line 176, 2.1.1 sub-group analysis, do you put the appropriate place?

   **Response**
   We appreciate your concern regarding the placement of Section 2.1.4 (Sub-group Analysis). After reviewing the structure, we confirm that this section is appropriately positioned within the manuscript. However, we have made minor adjustments to the arrangement to improve clarity and logical flow. We hope this revision enhances readability and aligns better with the overall structure of the paper.

8. Table 1, why not added the statistic, such as t, chi2, df and p-value?
    **Response**
   Thank you for your valuable insight. We have now included chi-square test statistics, including p-values in Table 1. Additionally, the interpretation of the chi-square results has been incorporated into the updated manuscript from lines 394–413 (as follows).

   “The median age of the sample population was 61 years, with smoking initiation occurring at a median age of 17 years (p < 0.001 and p = 0.003, respectively). Regarding cigarette consumption, individuals with high food security reported a median daily intake of 11 cigarettes, while those with marginal, low, and very low food security consumed a median of 10, 20, and 15 cigarettes per day, respectively. Daily cigarette consumption varied by food security status, with higher consumption among those with lower food security with a level of significance 0.040.

Among the 819 participants, males outnumbered females (432 vs. 387). The majority of both males (55%) and females (45%) had high food security; however, this difference was not statistically significant (p = 0.13). Severe depression was more prevalent among individuals with very low food security (28%), compared to those with marginal (4.5%) and low food security (15%), demonstrating a significant association (p < 0.001). Similarly, severe anxiety was significantly higher among participants with very low food security (27%) compared to those with high food security (7%) (p < 0.001).

In terms of physical health conditions, 8.9% of participants with very low food security had cardiovascular disease (p = 0.2). The prevalence of hypertension was similar among those with high (87%) and very low food security (84%) (p = 0.8). Among participants with diabetes, the prevalence was equal (22%) for those with high and very low food security (p = 0.5). Additionally, 42% of participants with low food security were obese, though this association was not statistically significant (p = 0.8). The marital status of participants showed mixed results with p-value of <0.001.”

9. Figure 1, the unit missed, difficult to see clear.

   **Response**
   Thank you for pointing this out. The histogram represents the number of cigarettes consumed daily. We have updated Figure 1 by adding the appropriate unit (number of cigarettes consumed in a day) to enhance clarity. The revised histogram has been incorporated into the manuscript.

We sincerely hope that the revised manuscript matches the journal’s standards. We sincerely appreciate the reviewers' and editors' time and thoughtful feedback, which have helped improve our manuscript. We have carefully addressed all comments and made the necessary revisions. Thank you for your consideration, and we look forward to your response.

Best regards,

Shafeel Umam,

Corresponding Author,

Department of Statistics and Data Science,

Jahangirnagar University, Savar, Dhaka-1342

Email: [shafeel.umam@outlook.com](mailto:shafeel.umam@outlook.com)

Rubaiya Binte Razzak,

Department of Statistics and Data Science,

Jahangirnagar University, Savar, Dhaka-1342

Email: [razzakrubaiya@gmail.com](mailto:razzakrubaiya@gmail.com)

Munniara Yesmin Munni,

PME department,

Christian Commission for Development in Bangladesh

Email: [ymunniara@gmail.com](mailto:ymunniara@gmail.com)

Azizur Rahman,

Department of Statistics and Data Science,

Jahangirnagar University, Savar, Dhaka-1342

Email: [rahman.aziz83@gmail.com](mailto:rahman.aziz83@gmail.com)

References:

1.	National Center for Health Statistics. National Health Interview Survey, 2022 survey description. [Internet]. National Center for Health Statistics.; 2023. Available from: https://ftp.cdc.gov/pub/Health_Statistics/NCHS/Dataset_Documentation/NHIS/2021/srvydesc-508.pdf

2.	National Center for Health Statistics. Advance letter. Available from: https://ftp.cdc.gov/pub/Health_Statistics/NCHS/Survey_Questionnaires/NHIS/2022/advance-letter-2022.pdf

3.	National Center for Health Statistics. National Health Interview Survey 2022 Capi Manual for NHIS Field Representatives. 2021 [cited 2024 Oct 3]; Available from: https://ftp.cdc.gov/pub/Health_Statistics/NCHS/Survey_Questionnaires/NHIS/2022/frmanual-508.pdf

4.	Kim JE, Tsoh JY. Cigarette Smoking Among Socioeconomically Disadvantaged Young Adults in Association With Food Insecurity and Other Factors. Prev Chronic Dis. 2016 Jan 14;13:E08.

5.	Kim-Mozeleski JE, Castele MC, Nambiar P, Chagin KM, Pike Moore S, Hardy P, et al. A Randomized Trial to Address Food Insecurity and Promote Smoking Cessation Among Low-Income Adults. J Prim Care Community Health. 2024 Jan 1;15:21501319241245275.

6.	Kim-Mozeleski JE, Seligman HK, Yen IH, Shaw SJ, Buchanan DR, Tsoh JY. Changes in Food Insecurity and Smoking Status over Time: Analysis of the 2003 and 2015 Panel Study of Income Dynamics. Am J Health Promot. 2019 Jun 1;33(5):698–707.

7.	Balinda IG, Sugrue DD, Ivers LC. More Than Malnutrition: A Review of the Relationship Between Food Insecurity and Tuberculosis. Open Forum Infect Dis. 2019 Apr 1;6(4):ofz102.

8.	Baek SU, Lee YM, Won JU, Yoon JH. Association of food insecurity with the use of tobacco products and urine cotinine-measured smoking intensity: evidence from a population-based study in South Korea, 2019–2021. Tob Control [Internet]. 2024 Oct 24 [cited 2025 Mar 3]; Available from: https://tobaccocontrol.bmj.com/content/early/2024/10/24/tc-2024-058754

9.	Robson SM, Lozano AJ, Papas M, Patterson F. Food Insecurity and Cardiometabolic Risk Factors in Adolescents. Prev Chronic Dis. 2017 Nov 9;14:E110.

10.	Kim-Mozeleski JE, Pandey R. The Intersection of Food Insecurity and Tobacco Use: A Scoping Review. Health Promot Pract. 2020 Jan 1;21(1_suppl):124S-138S.

11.	van der Velde LA, Nyns CJ, Engel MD, Neter JE, van der Meer IM, Numans ME, et al. Exploring food insecurity and obesity in Dutch disadvantaged neighborhoods: a cross-sectional mediation analysis. BMC Public Health. 2020 Apr 28;20(1):569.

12.	van den Brand FA, Candel MJJM, Nagelhout GE, Winkens B, van Schayck CP. How Financial Incentives Increase Smoking Cessation: A Two-Level Path Analysis. Nicotine Tob Res. 2020 Jan 29;23(1):99–106.

13.	Kang SY, Cho HJ. Association Between the Use of Tobacco Products and Food Insecurity Among South Korean Adults. Int J Public Health. 2022 Sep 8;67:1604866.

14.	Jo YH, Talmage DA, Role LW. Nicotinic receptor-mediated effects on appetite and food intake. J Neurobiol. 2002 Dec;53(4):618–32.

15.	Kim JE, Flentje A, Tsoh JY, Riley ED. Cigarette Smoking among Women Who Are Homeless or Unstably Housed: Examining the Role of Food Insecurity. J Urban Health. 2017 Aug 1;94(4):514–24.

16.	Sheira LA, Frongillo EA, Hahn J, Palar K, Riley ED, Wilson TE, et al. Relationship between food insecurity and smoking status among women living with and at risk for HIV in the USA: a cohort study. BMJ Open. 2021 Sep 1;11(9):e054903.

17.	Mayer M, Gueorguieva R, Ma X, White MA. Tobacco use increases risk of food insecurity: An analysis of continuous NHANES data from 1999 to 2014. Prev Med. 2019 Sep 1;126:105765.

18.	Bergmans RS. Food insecurity transitions and smoking behavior among older adults who smoke. Prev Med. 2019 Sep 1;126:105784.
