# Supplementary material for: Exploring the non-linear association of daily cigarette consumption behavior and food security- An application of CMP GAM regression
Source: PLoS One. 2025 Jul 17;20(7):e0328109. doi: 10.1371/journal.pone.0328109 (PMC12270119; doi:10.1371/journal.pone.0328109)
Supplement: Attachment — Submitted filename: Response_to_Reviewers_auresp_2.docx [file pone.0328109.s002.docx]

Dear Editor-in-Chief,

We, the authors of PONE-D-24-42111R1 titled “Exploring the non-linear association of daily cigarette consumption behavior and food security- An application of CMP GAM Regression,” appreciate the reviewer’s comments, who managed the time to examine this manuscript thoroughly and provided us with valuable insights. These suggestions greatly aided us in enhancing the manuscript, which has now been updated. We are confident that the content and clarity are significantly better in the revised version.

Response to the journal requirements are as follows:

**Journal Requirements:**

Please review your reference list to ensure that it is complete and correct. If you have cited papers that have been retracted, please include the rationale for doing so in the manuscript text, or remove these references and replace them with relevant current references. Any changes to the reference list should be mentioned in the rebuttal letter that accompanies your revised manuscript. If you need to cite a retracted article, indicate the article’s retracted status in the References list and also include a citation and full reference for the retraction notice.

**Response**

Thank you for your guidance regarding the reference list. We have carefully reviewed all cited references using both Zotero and EndNote citation management tools to identify any retracted articles. Based on this review, we confirm that there are no retracted articles included in our reference list.

Below are the responses to the reviewers’ comments. The source file (.docx) of the revised manuscript was attached in two forms: ‘with changes marked’ and our ‘revised manuscript.’

**Reviewer- 1**

Responses to the comments are provided below:

1. Report Median and IQR: For the variable "Daily Cigarette Consumption," include both the median and the interquartile range (IQR) if second column of Table 1. This provides a clearer picture of the central tendency and variability.

   **Response**
   Thank you for your helpful suggestion. We have included the median and the interquartile range (IQR) for “Daily Cigarette Consumption” in our manuscript. To enhance clarity, we have now also included this information explicitly in a footnote to Table 1 to ensure it is easily visible to readers.
2. Specify Indicators Clearly: For each variable, explicitly state which statistical indicators are being reported in front of the variable name in table 1. For example, for Daily Cigarette consumption you can use: Daily Cigarette consumption (Median, IQR).

   **Response**
   Thank you for this constructive feedback. To enhance clarity and maintain a clean presentation, we have specified the statistical indicators (e.g., Median and IQR for continuous variables such as age, smoking duration, and daily cigarette consumption) in a dedicated footnote to Table 1. This approach ensures consistency in interpretation while avoiding overcrowding the variable labels. A separate footnote is also provided for categorical variables, indicating frequencies and percentages (n, %).

We sincerely hope that the revised manuscript matches the journal’s standards. We sincerely appreciate the reviewers' and editors' time and thoughtful feedback, which have helped improve our manuscript. We have carefully addressed all comments and made the necessary revisions. Thank you for your consideration, and we look forward to your response.

Best regards,

Shafeel Umam,

Corresponding Author,

Department of Statistics and Data Science,

Jahangirnagar University, Savar, Dhaka-1342

Email: [shafeel.umam@outlook.com](mailto:shafeel.umam@outlook.com)

Rubaiya Binte Razzak,

Department of Statistics and Data Science,

Jahangirnagar University, Savar, Dhaka-1342

Email: [razzakrubaiya@gmail.com](mailto:razzakrubaiya@gmail.com)

Munniara Yesmin Munni,

PME department,

Christian Commission for Development in Bangladesh

Email: [ymunniara@gmail.com](mailto:ymunniara@gmail.com)

Azizur Rahman,

Department of Statistics and Data Science,

Jahangirnagar University, Savar, Dhaka-1342

Email: [rahman.aziz83@gmail.com](mailto:rahman.aziz83@gmail.com)
