# Supplementary material for: Exploring the non-linear association of daily cigarette consumption behavior and food security- An application of CMP GAM regression
Source: PLoS One. 2025 Jul 17;20(7):e0328109. doi: 10.1371/journal.pone.0328109 (PMC12270119; doi:10.1371/journal.pone.0328109)
Supplement: Attachment — Submitted filename: Response_to_Reviewers_auresp_3.docx [file pone.0328109.s003.docx]

Dear Editor-in-Chief,

We, the authors of PONE-D-24-42111R2 titled “Exploring the non-linear association of daily cigarette consumption behavior and food security- An application of CMP GAM Regression,” appreciate the reviewer’s comments, who managed the time to examine this manuscript thoroughly and provided us with valuable insights. These suggestions greatly aided us in enhancing the manuscript, which has now been updated. We are confident that the content and clarity are significantly better in the revised version.

Responses to the journal requirements are as follows:

**Journal Requirements:**

1) Please review your reference list to ensure that it is complete and correct. If you have cited papers that have been retracted, please include the rationale for doing so in the manuscript text, or remove these references and replace them with relevant current references. Any changes to the reference list should be mentioned in the rebuttal letter that accompanies your revised manuscript. If you need to cite a retracted article, indicate the article’s retracted status in the References list and also include a citation and full reference for the retraction notice.
    **Response**
   Thank you for your guidance regarding the reference list. We have carefully reviewed all cited references using both Zotero and EndNote citation management tools to identify any retracted articles. Based on this review, we confirm that there are no retracted articles included in our reference list.
2) While revising your submission, please upload your figure files to the Preflight Analysis and Conversion Engine (PACE) digital diagnostic tool, <https://pacev2.apexcovantage.com/>. PACE helps ensure that figures meet PLOS requirements. To use PACE, you must first register as a user. Registration is free. Then, login and navigate to the UPLOAD tab, where you will find detailed instructions on how to use the tool. If you encounter any issues or have any questions when using PACE, please email PLOS at [figures@plos.org](mailto:figures@plos.org). Please note that Supporting Information files do not need this step.

   **Response**

   Thank you for the instruction. We have uploaded our figure to the PACE digital diagnostic tool and reviewed the output to ensure it meets PLOS figure requirements. The revised figure, validated through PACE, has been incorporated into the updated manuscript accordingly. Please let us know if any additional adjustments are needed.

Below are the responses to the Additional Editor’s comments. The source file (.docx) of the revised manuscript was attached in two forms: ‘with changes marked’ and our ‘revised manuscript.’

**Additional Editor**

Responses to the comments are provided below:

1. For each variable in table 1, explicitly state which statistical indicators are being reported with the variable name. For example, for Daily Cigarette consumption you can use: Daily Cigarette consumption (Median, IQR).
   It is not suitable to make a note to describe in the table. The authors should revise the format according to the author’s guide.

   **Response**
   Thank you for the helpful suggestion. We have revised Table 1 to explicitly include the statistical indicators (e.g., *Median (IQR); n (%)*) for each variable name, as recommended. In making these changes, we followed the formatting structure used in Table 1 (on page 5) of a previously submitted and accepted PLOS ONE article titled "*Measure what matters: A survey-based examination of health equity tracking and measurement practices across healthcare systems in the United States*."(1) We hope this revised format now adheres to the PLOS ONE author guidelines and reflects consistency with the journal's published standards.

We appreciate your guidance and hope this revision addresses the concern.

We sincerely hope that the revised manuscript matches the journal’s standards. We sincerely appreciate the reviewers' and editors' time and thoughtful feedback, which have helped improve our manuscript. We have carefully addressed all comments and made the necessary revisions. Thank you for your consideration, and we look forward to your response.

Best regards,

Shafeel Umam,

Corresponding Author,

Department of Statistics and Data Science,

Jahangirnagar University, Savar, Dhaka-1342

Email: [shafeel.umam@outlook.com](mailto:shafeel.umam@outlook.com)

Rubaiya Binte Razzak,

Department of Statistics and Data Science,

Jahangirnagar University, Savar, Dhaka-1342

Email: [razzakrubaiya@gmail.com](mailto:razzakrubaiya@gmail.com)

Munniara Yesmin Munni,

PME department,

Christian Commission for Development in Bangladesh

Email: [ymunniara@gmail.com](mailto:ymunniara@gmail.com)

Azizur Rahman,

Department of Statistics and Data Science,

Jahangirnagar University, Savar, Dhaka-1342

Email: [rahman.aziz83@gmail.com](mailto:rahman.aziz83@gmail.com)

Reference:

1.	Roth H, Marchis ED, Kopaskie K, Restall A, Fichtenberg C, Ray S, et al. Measure what matters: A survey-based examination of health equity tracking and measurement practices across healthcare systems in the United States. PLOS ONE. 2025 May 21;20(5):e0323381.
